# Factors influencing non-conveyance care encounters in the ambulance service, registered nurses experiences - a qualitative study

**DOI:** 10.1186/s12912-024-01899-9

**Published:** 2024-04-24

**Authors:** L. Granlund, I Brännström, V Lindström

**Affiliations:** 1 Department of Ambulance Service, Region Västerbotten, Umeå, Sweden; 2 Department of Nursing, Umeå University, Umeå, Sweden

**Keywords:** Emergency medical services, EMS, Ambulance service, Registered nurses, Ambulance clinicians, Critical incident technique, Non-conveyance

## Abstract

**Background:**

There is a notable variation in the percentage of non-conveyed patients within the ambulance service. Discharging patients at the scene includes a risk of adverse events, and both patients and ambulance clinicians experience the complexity of non-conveyance. Therefore, this study aimed to describe factors influencing the care encounter when care in the ambulance service concludes with non-conveyance.

**Method:**

A qualitative study design employing the critical incident technique for data collection through individual interviews, and a qualitative analysis based on Fridlund et al. descriptions was utilized. The study conforms to the COREQ checklist for reporting qualitative research.

**Results:**

Fourteen Registered Nurses (RN) described 30 incidents and various factors were identified as influencing the care encounter. The factors included communication, sharing information, maintaining a secure and confident approach, organizational aspects, applying person-centered care in collaboration with the patient, relatives, and other caregivers, and an overall understanding of the patient’s entire situation. These factors were integrated into the RNs’ decision-making process for non-conveyance.

**Conclusion:**

The decision-making process for non-conveyance by RNs is a multifaceted approach that incorporates several factors. Communication, sharing of information, maintaining a secure and confident approach, organizational aspects, applying person-centered care in collaboration with the patient, relatives, and other caregivers, and a comprehensive understanding of the patient’s entire situation. These findings have the potential to contribute to the development of guidelines supporting the RNs working in the ambulance service in their decisions regarding non-conveyance. Further research is needed on the patient’s and relatives’ perspective on non-conveyance otherwise, patient participation and partnership in person-centered care are not possible to achieve.

**Supplementary Information:**

The online version contains supplementary material available at 10.1186/s12912-024-01899-9.

## Introduction

In the ambulance service, the care and medical interventions provided by ambulance clinicians are characterized by care in no predetermined care environments, unpredictable situations, independent decision-making, and limited background information about the patient [1, 2]. Ambulance clinicians have unique autonomy and independence in their assessment, triage, and decision-making processes [2]. Consequently, the quality of patient care relies heavily on factors such as the clinicians’ competence, experience, organizational support, and adherence to guidelines [3, 4]. The ambulance assignments have increased over time [5–7] and the non-conveyed patients constitute a significant and expanding proportion (3.7–93.7%) of the total patients attended to and cared for by ambulance services [3, 8–10]. In care encounters ending with a non-conveyance decision, ambulance clinicians have recognized the complexity of the processes for non-conveyance decisions, involving a balancing act of various expectations and encountering challenges due to limited organizational support [11–14]. The non-conveyed decision also involves considerations such as whether patients can remain at home if they should be referred to another level of care, the use of alternative transportation methods besides the ambulance, or advising self-care [3, 15, 16]. Despite the known challenges associated with non-conveying patients, ambulance clinicians continue making decisions to refrain from transporting patients to healthcare facilities.

## Background

*“Non-conveyance is a term used to describe a 999 call to the ambulance service that results in a decision not to transport the patient to a health-care facility”* [17]. Most of the non-conveyed patients are dispatched with the highest priority [15], reasons are not fully clear but may indicate the complexity of assessing emergency calls [18] or not asking the right questions to the caller [19]. Regardless, there is an increased demand for ambulance services that are not related to acute illness or injury [6, 7, 20, 21]. This can be attributed to several factors, the care seekers have a lower threshold for calling the emergency number [10], an aging population has increased emergency care needs [22], the accessibility to ambulance service has improved while health literacy has decreased among the care-seekers [7] and in addition, the ambulance services can provide assessment and care that includes alternative care pathways excluding the emergency department as a final destination [23]. However, the increased number of ambulance assignments and non-conveyance risks compromising accessibility, quality, and patient safety, and it may have an impact on the patient outcome and the care encounter. In the literature, there is a great variation (3.7–93.7%) in non-conveyed patients attended to and cared for by ambulance services [3, 8–10]. Factors influencing the variation in non-conveyed rates are described to stem from both controllable and uncontrollable factors within or outside the ambulance service [24]. The differences in non-conveyance rates can also be caused by different ways of defining, measuring, and presenting frequencies on non-conveyance. However, a factor known to increase the likelihood of a non-conveyance decision is when the ambulance arrives during the evening or night, and when the distance to a healthcare facility is between 21 and 40 km [25]. This could suggest, even though not known conclusively, either a shortage of nearby healthcare facilities capable of managing patients with minor illnesses or injuries during nighttime or limited ambulance resources in rural areas. Additionally, culture, leadership, and guidelines may also influence the frequency of non-conveyance [24] but to what extent is unclear. It is known that the initial complaints, conditions, symptom presentation, and age vary broadly among non-conveyed patients [3, 8, 15, 26]. It is also shown that non-conveyed patients have a risk of being exposed to adverse events [3, 10, 27, 28] and the non-conveyed patients often seek medical care within 72 h after assessment made by ambulance clinicians [29]. The patients’ experiences of being non-conveyed are described as ‘complex and versatile’ [30], satisfying by being empowered in the decision-making, or as negative experiences when concerns were not confirmed by the ambulance clinician [31, 32].

In conclusion, discharging patients at the scene includes a risk of adverse events, and both patients and ambulance clinicians experience the complexity of non-conveyance. Considering this, the study aimed to describe factors influencing the care encounter when care in the ambulance service concludes with non-conveyance. By delving into narratives on the phenomenon of non-conveyance described by Registered Nurses (RN) working in the ambulance service factors, challenges, and implications associated with non-conveyance can be clarified and support for ambulance clinicians and patients may be developed systematically.

## Methods

A qualitative study design was used to describe factors influencing the care encounter when care in the ambulance service concludes with non-conveyance. The study conforms to the COREQ checklist for reporting qualitative research [33].

### Study setting

The study was conducted in the Region of Västerbotten, located in northern Sweden, with an approximate population of 276,000 inhabitants. This region comprises both rural areas and cities. The ambulance service, which consists of 22 emergency ambulances and one helicopter, covers an area of 55,186 km². Annually, there are approximately 36 000 ambulance assignments, and the rate of non-conveyance is approximately 28%. However, the non-conveyance rate varies among different areas within the region. The ambulances are staffed with RNs who hold either a bachelor’s degree or an RN with an additional year of training in emergency care at an advanced level (second cycle training), which includes a one-year master’s degree. The RNs works with each other or with an emergency medical technician (EMT) possessing basic life support competencies. The assessment of patients made by the RNs and subsequent decision-making rely on supporting guidelines and the possibility of consulting a physician via telephone.

### Participants

The study’s inclusion criteria were RNs with firsthand experiences of care encounters, including non-conveyance within the ambulance services. The head of the department approved the study before inviting eligible participants to participate in the study. The written information about the study was distributed to eligible participants (*n* = 140) through local managers and a web service accessible to all eligible participants within the ambulance service in the region. Given that Critical Incident Technique (CIT) was utilized for data collection, the participants were determined based on the number of incidents rather than the number of individuals. Consequently, the inclusion of participants occurred consecutively, aiming for a balanced representation concerning gender, age, and years of service in the ambulance service. Two participants agreed to participate but declined without any reason before the interviews were conducted, none of the remaining participants requested to withdraw or discontinue their participation after the interviews were done. Following K FitzGerald, NS Seale, CA Kerins and R McElvaney [34] descriptions, saturation was acknowledged when participants’ descriptions of incidents stopped to yield additional insights and predominantly reflected repetitions. In total, from different geographical areas within the region fourteen participants (8 male and 6 female), age 27–60 years (mean 41.9), and with 1–36 years (mean 12.6) of working experience from the ambulance service collectively contributed 30 incidents for analysis.

### Data collection

Semi-structured individual interviews using CIT were utilized for data collection during spring 2023. CIT for data collection was selected as it allows participants to describe factors that either supported or hindered them [35] during care encounters that concluded with a non-conveyance. In accordance with CIT [35, 36], the participants were provided with the interview questions in advance to allow them to prepare by recalling and remembering care encounters that have been concluded with non-conveyance. The first and second authors conducted and recorded the interviews, and the interviews were carried out using a digital platform (*n* = 5) or at locations chosen by the participants (*n* = 9). The concept of critical incidents was not used during the interviews instead, the participants were encouraged to describe situations where the care encounter concluded with a non-conveyance that had felt particularly well/or not. All interviews started with demographic questions (age, gender, working experience, education) thereafter the participants were encouraged to describe their experienced care encounters/situations. Additional and follow-up questions to the narratives were, *What factors do you think contributed/influenced the care encounter, How was the situation/environment, What happened, What thoughts and emotions arose for you when the patient was non-conveyed, Could you elaborate further, Can you provide an example?* The questions used were developed by the authors for this study (see Supplementary file). The order of questions depended on the content and which questions matched the participants’ narratives. Two pilot interviews were conducted to ensure that the participants understood the questions and that the responses were complete and meaningful for CIT analysis, the pilot interviews are not included in the study. The interview lasted between 7 and 22 min (mean 15 min; total time of interviews 3 h, 55 min) minutes and was transcribed verbatim by first and second author.

### Analysis

Analysis was undertaken using an inductive qualitative approach as described by B Fridlund, M Henricson and J Mårtensson [37]. This analysis was chosen as it intends to identify and describe factors, with limited room for the authors to interpret the participants descriptions and answers to questions [37]. The analysis started with repeated readings of the transcribed text to establish familiarity with its content. Subsequently, factors associated with non-conveyance were color-coded, condensed, labeled, and extracted into an Excel spreadsheet. Thereafter, at a descriptive level, the extracted experiences were grouped, yielding a total of seven sub-categories. These sub-categories, sharing common content, were further organized into three main categories. Each step in the analysis process was initiated by one of the researchers and subsequently reviewed by the other researchers. Any differences in interpretation during the analysis were resolved through discussions until a consensus was reached among all researchers. During the whole analysis, a continuous movement between the transcribed text, color-coded extract, sub-categories, and main categories was done to preserve the experiences of non-conveyance described by the participants.

## Results

Three main categories and seven sub-categories emerged from the analysis: The interpersonal interaction in the care encounter, Optimizing care by non-conveyance, and External challenges in non-conveyance, as displayed in Table 1.

**Table 1:** Main categories and sub-categories

Main Categories	Sub-Categories
The interpersonal interaction in the care encounter	• The significance of information
	• The nurses’ approach and communication
	• The nurse’s security in their assessment
Optimizing care by non-conveyance	• Collaboration with the patient
	• Understanding the patient’s and their relative’s comprehensive situation
External challenges in non-conveyance	• The workplace environment
	• Inaccessible healthcare

### The interpersonal interaction in the care encounter

The main category *interpersonal interaction in the care encounter* refers to activities and communications between RNs, patients, relatives, and other professionals during the care encounter. The interpersonal interactions in the care encounter were influenced by various factors such as information, communication skills, collaboration, assessment, sense of security, and the RN approach, all factors influencing the outcomes for the patient, relatives, and the RNs deciding on non-conveyance.

#### The significance of information

In accordance with the RNs’ descriptions information was identified as a factor in shaping how the care encounter ended. When patients and relatives understood the provided information, RNs noted that they became reassured. Through information dissemination, the RNs perceived that the patients gained insight into their decision-making and understood the rationale behind the actions and decisions taken by the RNs. Furthermore, when RNs shared their knowledge with the patients, the patients showed comprehension. As a result, the patients expressed emotions regarding the actions and decisions made were able to shift from a negative experience of the care encounter to a positive experience.

*“When I suggested the patient use her usual way of transport to the hospital, she initially resisted, but then when I explained that it’s what we usually do when there’s no need for care and treatment during the ambulance transport, but she needs to go to the hospital, but we [ambulance] are not the best solution of transport, she understood and agreed it was great because then she could bring her wheelchair to the hospital. The information and explanations about the reasons for not being transported by the ambulance turned her emotions around and made it positive.”* Participant 2.

The RNs acknowledged that patients could experience anxiety or concern when a non-conveyance decision was made, but the RNs could succeed in comforting the patients by sharing knowledge and information regarding the patient’s symptom presentation and medical condition by explaining vital signs and symptoms. Ensuring patient comprehension of the conveyed information and providing them with the opportunity to ask questions were emphasized as a factor to reassure the patient and their relatives when deciding to non-convey the patient. The RNs perceived those patients presented trust in them when they shared knowledge and information. The RNs also described care encounters in which patients, or their relatives were unwilling to accept or failed to comprehend the provided information. Such disagreements and misunderstandings resulted in a negative experience in the care encounter.

*“The patient had decided that he wanted and needed emergency care and that my colleague and I couldn’t decide if the symptoms are acute or not. The patient felt that the illness still was severe and acute… we have different understandings of what the symptoms were caused by. Often, this kind of disagreement leads to a dissatisfied patient.”* Participant 7.

#### The nurses’ approach and communication

The approach taken by the RNs in the interpersonal interactions with patients and their relatives was described as a factor that influenced the care encounter. The RNs’ approach was also described as influencing the outcome of non-conveyance both positively and negatively. Creating a sense of security, fostering trust, and ensuring patient comfort in the care encounter were described by the RNs as factors influencing communication. Without these factors, the patients hold back from sharing their concerns, seeking information, or being reassured altogether resulting in a misunderstanding in communication. Devoting time to patients through attention was also described as a factor that positively influenced the care encounter. Similarly, the RNs described that comprehending the patients’ situation and offering validation and acknowledgment led to the RNs’ assumption that the patients felt heard and respected. Overall, when the RNs sensed that patients were being actively listened to, trusted, and treated seriously, the care encounter ended with a non-conveyance that was perceived as positive.

*“Not being stressed, I think. Listening to the patient, letting them talk. Many patients need to talk a lot, so just listening at first and acknowledging that I hear what you’re saying, I see that you’re in pain.”* Participant 4.

Communication challenges with the patients and their relatives were described to be caused by factors such as cultural differences, language barriers, or difficulties in reaching the patients through information. In addition, factors like stress, patients’ discomfort, and the time of day were identified as possible obstacles to achieving effective communication. Additionally, patients’ limited comprehension of the decision on non-conveyance also played a role in the dynamic of communication. The RNs described that involvement in the decision-making together with patients, their relatives, and other professionals was considered a contributing factor to a secure care encounter, ending without the need for conveyance.

*“All decisions regarding staying at home should be made in consultation with the patient, and the relatives present with them. It should feel right and appropriate for everyone involved.”* Participant 5.

#### The nurse’s security in their assessment

The RNs’ sense of security and confidence in assessing the perceived illnesses and symptom presentations of patients was described as a factor influencing their decisions regarding non-conveyance and shaping the outcome of the care encounter. The RNs described how they noted that patients perceive credibility when RNs showed confidence in their assessment, examination, and in interpretation of the situation. A factor that supported the RNs’ sense of security was the accuracy when using the systematic patient assessment. Possessing confidence in one’s professional competence and having experience in decision-making regarding non-conveyance were also identified as factors influencing the care encounter. Additionally, professional competence and experience were described to be advantageous when evaluating patients with established medical conditions that don’t always necessitate further care, for example, patients with diagnosed diabetes suffering from hypoglycemia.

*“I think a lot of it has to do with my professional role. If I feel confident in my assessment and my knowledge of the situation, I can express that to the patient. […] We have a much greater understanding than the general public, and that’s what matters, communicating basic knowledge to them to make them feel secure.”* Participant 1.

Another factor that influenced the care encounter and created a sense of security for the RNs was the collaboration with fellow ambulance clinicians and other healthcare professionals. Seeking assistance and validating the appropriateness of decisions regarding non-conveyance, as well as referring patients to self-care advice or a different level of care, were considered supportive. Furthermore, having access to medical consultation was perceived as a supportive factor, particularly in situations where the RNs, patients, and their relatives held different perspectives on required care. However, in cases where RNs and medical consultations had disparate views on patients’ care needs, the collaboration became challenging and could diminish the RNs’ confidence in patient assessment and create uncertainty in decision-making.

### Optimizing care by non-conveyance

Optimizing healthcare for the patient was central, and part of that involved identifying the appropriate level of care. Patient knowledge and the healthcare relationship were also described as essential aspects of the care. Meeting the patient in their home was advantageous as it allowed the nurse to gain a comprehensive understanding of the patient’s situation. Together with the patient and their relatives, the nurse could then develop an optimal care plan.

#### Identifying the appropriate level of care in collaboration with the patient

According to the descriptions provided by the RNs, a factor influencing the care encounter and their decisions on non-conveyance was the desire to aid the patient in the most appropriate level of care.

*“…we could assist the patient by arranging an appointment for her. Many patients have difficulty scheduling urgent appointments at primary care centers. But we could call a nurse at the primary care center and book an appointment there… It feels good. The patient doesn’t need to wait at the emergency department in the evening…. There is an increased risk when the emergency department sends someone home at 3–4 in the morning.”* Participant 5.

Furthermore, when patients did not want to go by ambulance, the possibility of administering treatment at the scene was identified as a factor that was positive for the care encounter and supported the decision for non-conveyance. The ability to formulate a care plan through collaboration with the patient and other healthcare professionals was another factor that facilitated decisions for non-conveyance. Moreover, the RNs’ sense of positivity from tailoring the level of care according to patients’ situations and preferences was also a factor influencing the care encounter and their decisions.

*“When I can help the patient to stay at home, it becomes so much better, in this case, the primary care nurse and a physician made a home visit during the same afternoon. And it turned out as well as possible for a patient who, in any other case, would have been forced to go to the hospital. But it took us 2.5 hours to arrange things, but it felt more dignified for the patient "* Participant 11.

According to the RNs’ descriptions, another factor that influenced their ability to optimize care for patients through non-conveyance was to establish collaborations with various professionals within the municipality. The collaborations supported for example arrangements for extended home care or assistance in locating housing when faced with an unsuitable home environment.

*“…. I managed to reach the social worker for this woman who arranged a place in a municipal facility for respite care on the same day.”* Participant 11.

#### Understanding the patient’s and their relative’s comprehensive situation

Factors that influenced the care encounter and supported decisions about the necessity of care, conveyance, or non-conveyance were described as understanding the patient’s and their relative’s comprehensive situation, encompassing their personality and needs. Additionally, working within a small community facilitated an in-depth knowledge of the healthcare structure, available resources within the community, the patient’s health history, prior healthcare visits, and the patient’s social context. In some cases, outlined by the RNs, both patients and their relatives were present during the care encounter. Additionally, in some of the cases, it was the relatives who contacted the ambulance service based on their perception of a care requirement, even if the patients themselves didn’t share the same viewpoint or were hesitant to acknowledge their needs. Regardless, when relatives were present the care included the patient along with their relatives and the relatives became integral to the care and a factor influencing the care encounter and the RNs decision-making.

“*The patient wasn’t bothered it was the wife who took control. She often advocated for the patient even when it wasn’t necessary…I needed to tell the wife that the husband needed to tell his own story, how he felt, and how he perceived the situation… It didn’t go well because she got a little angry with us.”* Participant 3.

The expectations of both patients and their relatives upon calling the emergency number and receiving an ambulance were a factor that had an impact on the care encounter ending with a non-conveyance. When the RNs had made their assessment of the patient and decided to non-convey the patient by ambulance to a healthcare facility the relatives and patients sometimes reacted with frustration. On the other hand, some patients and their relatives were very understanding and positive about the care options or care plans described by the RNs. Regardless, to reassure the relatives the RNs informed both patients and their relatives aiming for a mutual understanding of the knowledge that emergency care and transport by an ambulance are not always the best solution for the patient.

### External challenges in non-conveyance

The factors identified as challenging in the care encounter and the decision on non-conveyance were those beyond the nurses’ control, including the working environment, limited healthcare resources, and an increased number of ambulance assignments.

#### The workplace environment

The ambulance assignments varied throughout the shift, and the nurses described a substantial workload, frequently with limited or no chances for recovery. The increase in ambulance assignments affected the RNs’ working environment negatively and was described as contributing to exhaustion that could result in a decrease in both the patience and educational effectiveness of RNs. Consequently, according to RNs, this may harm patient care by eroding trust and impeding communication between the RNs and the patient and or the relatives.

“*At times, when fatigue sets in or after extended periods of driving, one’s ability to be pedagogical may diminish. This poses a risk of eroding the trust that has been built* [with the patient]. *There may not be the capacity or time to rebuild it, and as a consequence, the outcome may not be as favorable* [for the patient].” Participant 8.

#### Inaccessible healthcare

Another factor influencing the care encounter and the decision to non-convey patients was the difficulties RNs faced when trying to schedule appointments at primary healthcare centers (PHCs). This led to frustration among RNs and was by the RNs identified as a problem stemming from resource shortages at PHCs. The issue was perceived as problematic both when patients independently sought appointments with PHCs and when RNs tried to schedule near-term appointments with PHCs for patients not in need of emergency care and who could be non-conveyed.

*” Currently, primary care centers provide a restricted number of appointments, typically scheduled days in advance. Securing emergency appointments for patients is rare due to frequent full bookings "* - Participant 5.

The shortage of PHC resources, along with a scarcity of available appointments for psychiatric care, were identified as problematic by the RNs. Consequently, some patients ended up in the emergency department even when it wasn’t the most suitable destination for their care or was non-conveyed without safety netting. The RNs concluded that the quality of care could be negatively affected when patients encountered difficulties accessing care at the appropriate level.

*Accessing primary care via phone is exceptionally challenging, leading to an increased reliance on ambulance services to assess patients’ care needs… The 1177* [telephone advice line] *experiences overload, with callers facing 45-minute waiting times and often being redirected to the 112* [emergency number]. resource*Unfortunately, 112’s only option is to dispatch an ambulance, even when emergency care is not initially necessary. Patients do not seek emergency care, but shortages in other healthcare services force them to 112, and thereby it becomes the ambulance service’s responsibility to address the issue of resource shortages in primary healthcare services* - Participant 12.

Another factor contributing to non-conveyance occurred when the RN contacted the responsible physician for urgent decisions regarding emergency care or compulsory psychiatric care, and the physician’s decision did not align with the RN’s assessment of the patient’s need for care. These situations were described as frustrating, as the nurses were present with the patients and recognized the imperative need for care and further actions. The RNs found it challenging and ethically uncomfortable to leave patients at home without support or additional plans for care or follow-up. Similar emotions emerged when patients chose not to accompany the ambulance, even when the RNs recognized the necessity for further care and strongly recommended it.

*“Convincing him to follow us to the hospital was impossible, and there were no grounds to detain him. He said that he had a gun at home, but I never saw it. When asked if he intended to harm himself or others, his response was elusive — a smile, as if to imply, ‘We’ll see…’ I tried to secure a compulsory psychiatric care decision by calling the psychiatric unit, but they dismissed that possibility, by stating, ‘It’s not possible.”* - Participant 11.

## Discussions

This study aimed to describe factors influencing the care encounter when care in the ambulance service concludes with non-conveyance. Overall, the identified factors influenced the care encounter both positively and negatively and were also integrated into the decision-making process for non-conveyance made by the RNs. The findings highlight the importance of communication, information sharing, and a secure and confident approach by nurses to ensure positive outcomes in the care encounter. This finding aligns with prior research describing the complex nature of non-conveyance in the ambulance service [11–14] But, to our knowledge, the factor of when the RNs shared their knowledge with the patients, and how this resulted in patients demonstrating comprehension, trust, and confidence in the non-conveyance decision is not as well described in the literature. Whether this relates to patients’ literacy and knowledge, or RNs’ competencies in sharing their knowledge is not known from this study’s findings. However, it is reasonable to assume that sharing knowledge and information is essential when non-conveyance is decided; otherwise, patient safety may be compromised. Regardless, it is known that communication is essential for gaining trust and increasing satisfaction among patients and communication should be viewed from both the patients’ and nurses’ perspectives, as the experience can significantly differ between them [38]. In this study, RNs’ view was explored but we need further knowledge about the patient’s perspective and relatives in case of non-conveyance, without knowledge of their perspectives, achieving person-centered care becomes challenging. In this study factors that were significant for the outcome of the care encounter included the need for person-centered care and care planning, collaboration with the patient, relatives, and other caregivers, as well as an overall understanding of the patient’s situation. This finding is in line with previous studies describing the complexity of non-conveyance [11–14]. However, in addition to previous studies, our study highlights the organizational factors that have an impact on the care encounter affecting both the patients and the RNs. These factors included shortages of resources in primary care services, sub-optimal opportunities for collaboration with other healthcare providers and stakeholders, and the shortage of streamlined processes of non-conveyance. The findings regarding organizational factors could possibly reflect that the participants in our study worked in rural areas with limited access to healthcare facilities. Previous studies on non-conveyance have not rural areas in the same extent as in our study context [3, 11–16]. Based on our study’s findings, our assumption is that it is crucial to enhance safety-netting for patients and increase, communication, and collaboration between the ambulance service and other healthcare services. This assumption maybe especially true in rural areas where there is limited access to healthcare facilities. However, research on safety-netting for patients who are non-conveyed by the ambulance service needs to be systematically developed and evaluated.

The increased demand for the ambulance service, coupled with greater complexity in care, has resulted in additional responsibilities for RNs. This is particularly evident in sub-acute care, psychiatric care, and assignments involving social distress, given the rising prevalence of these patients in ambulance services [39, 40]. In Sweden, there are no explicit demands within the ambulance service that RNs should decide on non-conveying patients. Our study and previous research show a lack of support for deciding not to convey [11–14] and still RNs take responsibility for this decision. Whether this reflects RNs’ desire to assist patients in reaching optimal levels of care or if they are acting as gatekeepers is not entirely clear, and further research into why RNs decide to non-convey is needed. However, in our study, the increased demand for the ambulance service was described by the RNs as influencing their decisions on non-conveyance. This could be an act of gatekeeping, which can be a part of non-conveyance decisions.

RNs may experience an increased sense of helplessness and frustration when they are unable to adhere to their standards due to shortages in primary care services and sub-optimal opportunities for collaboration with other healthcare providers. An assumption of this is that a sense of helplessness and frustration during care encounters may lead to heightened stress levels among RNs, and potentially increase the risk of patient safety issues as described by Al Ma’mari et al. [41]. This is supported by the knowledge that deciding on non-conveyance is a risk of compromising patient safety [3, 10, 27, 28]. Moreover, a working environment characterized by an imbalance of high-emergency and complex low-acuity assignments can impact the quality of life for ambulance personnel [42]. Beyond factors influencing the care encounter and the RNs’ decision-making on non-conveyance, the findings indicate a lack of common assessment criteria on patients’ need for care throughout the chain of care. Different healthcare providers may not share the same goals and conditions regarding where patients should be treated and cared for. Lack of knowledge about one’s own and others’ organizations can result in patients being referred between different healthcare providers without anyone taking responsibility, and the RNs in the ambulance service may also compromise patient safety when deciding on non-conveyance and if no safety netting is established. However, this is not answered in this study and further research is needed to explore and understand the phenomenon of non-conveyance and the need for safety netting in the ambulance service. Nevertheless, our findings and discussions together emphasize the importance of developing support systems for RNs who make decisions regarding the non-conveyance of patients. It also emphasizes the importance of education and consensus in the care where patients are involved in the decisions being made.

### Methodological considerations and limitations

A qualitative study design was selected to identify and describe factors influencing care encounters resulting in a decision on non-conveyance. The findings confirms previous studies and contributes with additional knowledge and understanding of the phenomenon of non-conveyance in ambulance services. But, as discussed, we still do not have a full comprehension and knowledge of the underlying factors that may influence the decisions on non-conveyance in ambulance services. However, the findings in this study should be interpreted with the following limitations. Firstly, the number of participants and the amount of CIT can be considered low. Nevertheless, the participants were well-balanced in terms of gender, age, and years of work in the ambulance service. Additionally, during the interviews, the CIT presented by the RNs became repetitive after interview number 11. Secondly, the length of interviews could be considered short. A reason for the relatively short interviews could be that the care encounters described by the participants were brief and concise. However, during the analysis, the descriptions were regarded as rich and varied. To improve and strengthen the credibility of the findings, additional participants could have been included. A more exploratory approach in the interviews might have provided a more comprehensive understanding of the factors influencing care encounters resulting in a non-conveyance decisions. The third limitation of this study may have been the used analysis, a thematic analysis could have been a better solution, as it can be applied across a range of theoretical and epistemological frameworks [35]. However, the chosen analysis was selected as it allowed minimal interpretation by the authors [37]. The fourth limitation is that all the authors are RNs, female, and possess extensive clinical experience in the ambulance service. This background might introduce biases during the study design, data collection, and analysis. Despite the potential for bias, the authors’ preconceptions can also be considered a strength as they contribute to interpreting and understanding the study findings. To minimize the risk of influencing the analysis, we followed Fridlund et al.‘s description of how to conduct the analysis. Additionally, we maintained a continuous movement between the transcribed text, color-coded extracts, sub-categories, and main categories. To include participants from only one county in one country may potentially limit the transferability of the findings. However, care in ambulance services is similar worldwide, characterized by relatively short patient encounters, acute and low-acute assignments, care in unpredictable situations, independent decision-making, and limited background information about the patient, as well as non-conveyance of patients. Therefore, it is reasonable to assume that the findings of this study can be transferable to similar contexts.

## Conclusion

Various factors were identified influencing the care encounter and the decision on non-conveyance, as perceived by the RNs. The factors included communication, sharing information, maintaining a secure and confident approach, applying person-centered care in collaboration with the patient, relatives, and other caregivers, as well as an overall understanding of the patient’s entire situation. These findings have the potential to enhance the care encounter and contribute to the development of guidelines supporting the RNs working in the ambulance service in their decisions regarding non-conveyance for patients seeking care by calling the emergency number and receiving an ambulance. However, more research is needed on the patient’s and relatives’ perspective on non-conveyance and possible need of safety netting otherwise, patient participation and partnership in person-centered care are not possible to achieve.

### Electronic supplementary material

Below is the link to the electronic supplementary material.

Supplementary Material 1

## Data Availability

The data analyzed during the current study are available from the corresponding author on reasonable request.
