# Supplementary material for: Factors influencing non-conveyance care encounters in the ambulance service, registered nurses experiences - a qualitative study
Source: BMC Nurs. 2024 Apr 24;23:271. doi: 10.1186/s12912-024-01899-9 (PMC11044363; doi:10.1186/s12912-024-01899-9)
Supplement: Supplementary file 1 — Supplementary Material 1 [file 12912_2024_1899_MOESM1_ESM.docx]

Supplementary file

Interview guide used in the study Exploring Non-Conveyance in Ambulance Services: Nurses' Descriptions of Care Encounters - A Qualitative Study

The authors developed the questions used and all the interviews started with demographic questions.

What is your age, gender, working experiences in the ambulance service, and previous education at university level?

Thereafter the participants were encouraged to describe their experienced care encounters/situations including non-conveyance.

Additional and follow-up questions to the narratives were:

*What factors do you think contributed to/influenced the care encounter?*

*How was the situation/environment in the care encounter?*

*What happened?*

*What thoughts and emotions arose for you when the patient was non-conveyed?*

*Could you elaborate further?*

*Can you provide an example?*
